# Methylation of Ribosomal RNA: A Mitochondrial Perspective

**DOI:** 10.3389/fgene.2020.00761

**Published:** 2020-07-17

**Authors:** M. Isabel G. Lopez Sanchez, Miriam Cipullo, Shreekara Gopalakrishna, Anas Khawaja, Joanna Rorbach

**Affiliations:** ^1^ Division of Molecular Metabolism, Department of Medical Biochemistry and Biophysics, Karolinska Institutet, Solna, Sweden; ^2^ Centre for Eye Research Australia, Melbourne, VIC, Australia; ^3^ Max Planck Institute for Biology of Ageing – Karolinska Institutet Laboratory, Karolinska Institutet, Stockholm, Sweden

**Keywords:** mitochondria, RNA, ribosome, methylation, methyltransferases, epigenetics

## Abstract

Ribosomal RNA (rRNA) from all organisms undergoes post-transcriptional modifications that increase the diversity of its composition and activity. In mitochondria, specialized mitochondrial ribosomes (mitoribosomes) are responsible for the synthesis of 13 oxidative phosphorylation proteins encoded by the mitochondrial genome. Mitoribosomal RNA is also modified, with 10 modifications thus far identified and all corresponding modifying enzymes described. This form of epigenetic regulation of mitochondrial gene expression affects mitoribosome biogenesis and function. Here, we provide an overview on rRNA methylation and highlight critical work that is beginning to elucidate its role in mitochondrial gene expression. Given the similarities between bacterial and mitochondrial ribosomes, we focus on studies involving *Escherichia coli* and human models. Furthermore, we highlight the use of state-of-the-art technologies, such as cryoEM in the study of rRNA methylation and its biological relevance. Understanding the mechanisms and functional relevance of this process represents an exciting frontier in the RNA biology and mitochondrial fields.

## Epigenetic Modifications of rRNA

RNA modifications are present in all living organisms and play important roles in RNA metabolism. The number of experimentally identified RNA modifications is growing, and to date, more than 170 RNA modifications have been reported (Boccaletto et al., 2018). RNA modifications are predominantly found in transfer RNA (tRNA), with modifications identified in up to 20% of nucleotides (Jackman and Alfonzo, 2013). Although not as common as in tRNA, human cytosolic ribosomal RNA (rRNA) contains 14 distinct types of post-transcriptional modifications in 228 sites (Taoka et al., 2018), while *Escherichia coli* rRNAs contain 36 modified nucleotides (Sergiev et al., 2011). Among the different types of rRNA modifications, 2′-O-methylation of the ribose followed by pseudouridylation is the most common (for a review on this abundant RNA modification see Charette and Gray, 2000).

In bacteria, most of the methylated nucleotides in the small subunit (SSU) of the ribosome are located on the surface and are introduced during the late stages of ribosome assembly, while nucleotide modifications in the large subunit (LSU) occur during early stages of assembly (Siibak and Remme, 2010). In eukaryotes, the introduction of rRNA modifications is closely linked to rRNA processing events and coupled to various stages of ribosome assembly (Armistead et al., 2009; Bourgeois et al., 2015; Zorbas et al., 2015).

In most cases, the precise role of rRNA modifications remains unclear. Some rRNA modifications are located on highly conserved nucleotides and cluster in functionally important areas of the ribosome, including the peptidyl transferase center and decoding site (Decatur and Fournier, 2002; Polikanov et al., 2015). This suggests that they may play important roles, including alteration of ribosomal active sites and stabilization of the rRNA scaffold (Demirci et al., 2010b; Polikanov et al., 2015). Furthermore, the presence of partial modifications, including 2′-O-methylation in a subset of rRNAs, indicates that nucleotide modifications may play additional roles under different physiological conditions (Krogh et al., 2016). Alterations in rRNA modification patterns have also been described during development (Blanco and Frye, 2014), in response to environmental changes (Schwartz et al., 2014) and in disease (Armistead et al., 2009). While it remains unclear how rRNA modifications affect overall cellular function, it is becoming evident that rRNA modifications are dynamic factors in the regulation of gene expression and may contribute to the fine-tuning of translation regulation.

## Methylation of rRNA in Bacteria and the Eukaryotic Cytosol

### Universally Conserved rRNA Methylation Sites

Methylation of rRNAs is a ubiquitous feature in all living organisms, and the presence of methylated rRNA residues at corresponding sites in prokaryotes and eukaryotes indicates that it is evolutionarily conserved. There are two universally conserved methylated residues in the SSU, N^6^-dimethylated adenines m^6^_2_A1518 and m^6^_2_A1519 (*E. coli* 16S rRNA numbering) located in helix 45 (Poldermans et al., 1979). Methylation at these sites facilitates contact between helices 44 and 45 near the decoding center of the ribosome through the formation of a hydrogen-bonding network that stabilizes this contact site (Wimberly et al., 2000; Demirci et al., 2010b). Absence of this dimethylation results in the rearrangement of the ribosomal decoding center and decreases fidelity of translation initiation and elongation (Demirci et al., 2010b). Methylation at these sites is introduced by KsgA, a highly conserved methyltransferase present in almost all living organisms (Poldermans et al., 1979; Formenoy et al., 1994). DIMT1L, the mammalian homolog of KsgA, is responsible for dimethylation in helix 45 and also plays a role in the assembly of the small ribosomal subunit through its independent function in pre-rRNA processing (Zorbas et al., 2015). In mitochondria, corresponding modifications are introduced by TFB1M, described in detail in the “mt-SSU Methyltransferases” section below.

The LSU contains two universally conserved modified nucleotides, Gm2251 and Um2552 (*E. coli* 23S rRNA numbering), located in the P-loop and A-loop (helices 80 and 92, respectively; Kiss-László et al., 1996; Lövgren and Wikström, 2001). Structural analyses indicate that the Um2552 methylation intercalates between the adjacent bases G2553 and U2554, thus preserving the active conformation of the G2553 base, which is directly involved in accommodating the aminoacyl-tRNA (Polikanov et al., 2015). Similarly, the 2′-O-methyl group of Gm2251 forms hydrophobic contacts with C2065 and U2449 that maintain the active conformation of the nucleotides involved in base pairing with P‐ and A-site tRNAs (Polikanov et al., 2015). While the absence of Gm2251 in *E. coli* has no phenotypic effects (Lövgren and Wikström, 2001), the lack of Um2552 results in a significant accumulation of assembly intermediates of the LSU (Tan et al., 2002). In bacteria, Gm2251 and Um2552 are introduced by the methyltransferases RlmB and RlmE, respectively (Caldas et al., 2000; Lövgren and Wikström, 2001), while the human cytoplasmic equivalents, Gm4196 and Um4498, are catalyzed *via* a small nucleolar RNA (snoRNA)-guided mechanism (Kiss-László et al., 1996) and an unknown mechanism, respectively. In mitochondria, modifications corresponding to Gm2251 and Um2552 are introduced by mitochondrial rRNA methyltransferase 1 (MRM1) and MRM2 respectively (described below in “mt-LSU Methyltransferases” section).

### Enzymes Responsible for Methylation of rRNA

Methylation of rRNAs takes place during ribosomal biogenesis either by enzymes guided by an antisense snoRNA or by conventional protein enzymes. All enzymes responsible for known rRNA methylation sites in *E. coli* and the yeast, *Saccharomyces cerevisiae*, have now been identified (Sharma and Lafontaine, 2015). However, the enzymes responsible for the modification of nucleotides Um4498, Gm4499, and m^3^U4530 in humans remain to be identified.

In eukaryotes, the most common rRNA modifications, 2′-O methylation and pseudouridylation, are catalyzed by small nucleolar ribonucleoprotein (snoRNP) particles that consist of snoRNA and proteins and occur simultaneously with the processing of rRNA precursors (Phipps et al., 2011). snoRNAs act as guides for snoRNPs *via* sequence complementarity with their respective rRNA target sequence (Reichow et al., 2007). Most snoRNPs fall into two large categories, C/D and H/ACA snoRNPs; C/D snoRNPs mediate 2′-O methylation, while H/ACA snoRNPs are responsible for pseudouridylation modifications (for a review see Watkins and Bohnsack, 2012). Other modifications are catalyzed by methyltransferases that modify specific rRNA nucleotides and do not require the participation of snoRNPs. To date, 57 RNA methyltransferases have been identified in humans (Schapira, 2016). With rare exceptions (Lesnyak et al., 2006; Kimura et al., 2012), each methyltransferase is responsible for the methylation of one rRNA nucleotide only.

In addition to methylation, several rRNA methyltransferases are involved in other aspects of ribosomal biogenesis, including pre-rRNA processing (Armistead et al., 2009). Interestingly, it has been shown that the role of some methyltransferases in pre-rRNA processing may be more critical to cellular function than their role in modifying rRNA. This is likely explained by the fact that eukaryotic 5.8S, 18S, and 28S rRNAs are encoded by a single, long polycistronic transcript that requires extensive processing by multiple assembly factors, including RNA-modifier enzymes, to release mature rRNAs (Kressler et al., 1999a). The existence of pre-rRNA processing enzymes that also function as methyltransferases may thus reflect a quality control mechanism, whereby methylation of certain rRNA nucleotides is dependent upon the generation of mature rRNAs.

## Biological Significance of rRNA Methylation

Numerous studies have shown that methylation of rRNA may have important implications for human health. This is primarily due to its role in antibiotic resistance, a potential role in cancer development, and because of genetic diseases caused by mutations in the rRNA methylation machinery components.

### Antibiotic Resistance

Most ribosome-targeting antibiotics interact exclusively with bacterial rRNA. Bacteria have evolved several mechanisms of resistance to antibiotics, including through the methylation of specific rRNA nucleotides that prevents the binding of protein synthesis inhibitors to their target sites on the bacterial ribosome. For instance, N^1^ methylation of A1408 in the bacterial 16S rRNA confers resistance against aminoglycosides (Kanazawa et al., 2017). Loss of methylation can also decrease antibiotic sensitivity. A classic example of this is the lack of methylation at A1518 and A1519 in 16S rRNA by KsgA, which confers resistance to kasugamycin (Poldermans et al., 1979). Similarly, the loss of m^2^A2503 in 23S rRNA, catalyzed by RlmN, confers resistance to antibiotics that target the peptidyl transferase center of the ribosome (Stojković et al., 2016). These examples highlight the important role of methylation in regulating the response to antibiotics.

### Cancer

There is increasing evidence linking messenger RNA (mRNA) or tRNA methylation and cancer. For instance, the m^6^A modification in mRNAs is associated with tumor proliferation in endometrial cancer (Liu et al., 2018), while m^5^C methylation of tRNAs by NSUN2 in skin cancer cells has been associated to tumorigenesis (Blanco et al., 2016). Similarly, altered ribosome biogenesis has been associated with the development of various cancers (Truitt and Ruggero, 2016). Evidence linking rRNA methylation and cancer comes from the inactivation of the tumor-suppressor gene *p53*, which resulted in an altered rRNA methylation pattern (Marcel et al., 2013). Future studies may elucidate the role of individual rRNA modifications in cancer. This is of particular interest given that modulation of ribosome biogenesis may also provide an alternative mechanism to arrest cell proliferation and delay tumor formation (Brighenti et al., 2015).

### Pathogenic Mutations in Methyltransferases

There is a growing list of human genetic disorders named ribosomopathies that are caused by mutations in genes encoding ribosomal proteins or ribosome biogenesis cofactors, including those involved in the rRNA methylation machinery. For instance, a point mutation in the EMG1 methyltransferase causes Bowen-Conradi syndrome, a ribosomopathy characterized by severe developmental delay and growth failure that often leads to early infant death (Armistead et al., 2009). Prader-Willi syndrome, a neurological disease characterized by intellectual disability, obesity, and muscle hypotonia is caused by deletions in the locus 15q11–q13, which contains a cluster of snoRNAs involved in RNA 2′-O-methylation (Sahoo et al., 2008). Similarly, mutations in the family of NOL1/NOP2/sun (Nsun) domain-containing genes encoding RNA methyltransferases in humans are associated with neurodevelopmental disorders (Blanco and Frye, 2014). However, due to the dual role of some methyltransferases in pre-rRNA processing, the exact contribution of impaired rRNA methylation to the pathology of these disorders requires further investigation.

## Mitochondrial RNA Expression

Evolutionarily originated from α-proteobacteria that were engulfed by a primitive cell (Roger et al., 2017), mitochondria retain their own circular double-stranded DNA along with their own protein translational machinery. Mammalian mitochondrial DNA (mtDNA) is 16,569 bp and is maternally inherited (Kaneda et al., 1995). It encodes a total of 37 genes, including 2 rRNAs, 22 tRNAs, and 13 polypeptides of the oxidative phosphorylation (OxPhos) system (Anderson et al., 1981). mtDNA exists as compactly-packed nucleoid structures of ~100 nm with mitochondrial transcription factor A (TFAM) being the core packaging factor (Brown et al., 2011; Kukat et al., 2011).

Unlike their cytosolic counterparts, mitochondrial RNAs (mt-RNAs) are transcribed as long polycistronic transcripts and require endonucleolytic cleavage for individual transcripts to be released. Processing of mitochondrial transcripts flanked by mitochondrial tRNAs (mt-tRNAs) involves cleavage by Ribonuclease P (RNaseP) complex and ElaC Ribonuclease Z 2 (ELAC2; Holzmann et al., 2008; Brzezniak et al., 2011; Sanchez et al., 2011), while mitochondrial transcripts that are not flanked by mt-tRNAs require additional protein factors for processing, including FASTKD4, FASTKD5, and GRSF1 (Jourdain et al., 2013, 2017). Similar to their cytosolic counterparts, mt-RNAs also are polyadenylated; however, the poly(A) tails are shorter, with an average length of 45–55 nucleotides, while the ND6 transcript is not polyadenylated at all (Temperley et al., 2010). Polyadenylation of the 3′ end of mt-RNAs is essential for the completion of stop codons of several mitochondrial transcripts and, therefore, for correct translation of their open-reading frames. Mutations in poly(A) polymerase (mtPAP) have been linked to neurodegenerative disease (Crosby et al., 2010).

Mitochondria maintain their own ribosomes (mitoribosomes) and translation system. The mammalian mitoribosome consists of RNA and proteins, with 16S mitoribosomal rRNA (mt-rRNA) and a mt-tRNA belonging to the mitoribosome large subunit (mt-LSU), and 12S mt-rRNA belonging to the mitoribosome small subunit (mt-SSU). There are 82 mitoribosomal proteins (Table 1), 36 of which are mitochondria-specific, while many proteins with homologs in bacteria have mitochondria-specific extensions.

**Table 1:** Composition of eukaryotic and prokaryotic small and large ribosomal subunits.

Ribosome	Monosome sedimentation rate	Small subunit	Large subunit
Eukaryotic	80S	40S: 18S rRNA and 33 ribosomal proteins (mammals)	60S: 5S, 5.8S, and 28S (mammals) rRNAs and 47 ribosomal proteins (mammals)
Prokaryotic	70S	30S: 16S rRNA and 22 ribosomal proteins	50S: 5S and 23S rRNAs and 34 ribosomal proteins
Mitochondrial	55S	28S: 12S rRNA and 30 ribosomal proteins	39S: mitochondrially-encoded tRNA, 16S rRNA, and 52 ribosomal subunits

Although mitoribosomes are similar to their bacterial counterparts, there are some key differences. For instance, while the RNA:protein ratio in bacterial ribosomes is 2:1, it is 1:2 in mitoribosomes, due to the large rRNA reductions and recruitment of new proteins stabilizing the mitoribosomal structure (Mears et al., 2006). Structural studies of the mammalian mitoribosome revealed that 5S rRNA is absent from the central protuberance of the mt-LSU. Instead, a mitochondrially-encoded tRNA^Val^ was detected in human and tRNA^Phe^ in porcine mitoribosomes (Amunts et al., 2015; Greber et al., 2015). Another significant adaptation of mitoribosomes is the presence of mitochondria-specific proteins with highly hydrophobic amino acid residues facing the ribosomal exit tunnel, due to the hydrophobic nature of the mtDNA-encoded OxPhos subunits (Amunts et al., 2015; Greber et al., 2015).

## RNA Modifications in Mitochondria

Numerous nuclear-encoded enzymes have been shown to introduce a wide range of modifications on mt-tRNAs. To date, 15 different types of modifications have been detected at 118 positions in mt-tRNAs, some of which occur within the anti-codon loop and are important for tRNA decoding, while others are important for the stabilization of tRNA structures and their recognition by aminoacyl-tRNA synthetases (reviewed in Suzuki and Suzuki, 2014). In contrast, while recent findings reported the presence of multiple pseudouridine and m^1^A sites in mt-mRNAs (Carlile et al., 2014; Antonicka et al., 2017; Li et al., 2017; Safra et al., 2017), their importance in the regulation of mitochondrial gene expression still needs to be elucidated.

**Figure 1:**
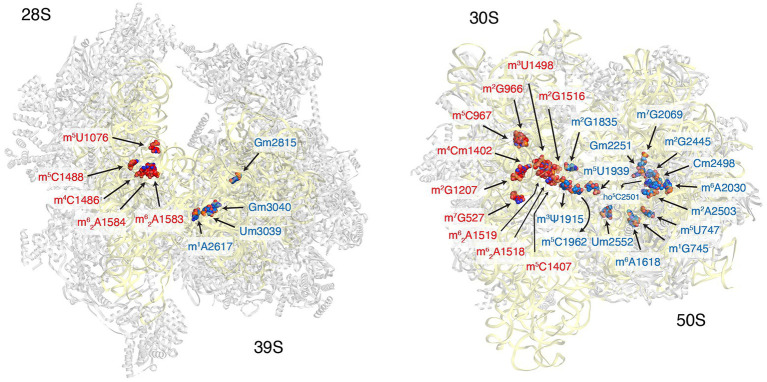
Distribution of ribosomal RNA (rRNA) methylation sites on human mitoribosome and bacterial ribosomes. The location of rRNA-methylation sites on ribosomal small subunit (red) and large subunit (blue) are displayed on the structure of human mitoribosome (left, PDB: 3J9M) and *Escherichia coli* (right, PBD: 4YBB). The ribosomal proteins are colored gray and the rRNA is in yellow.

The total number of modifications mapped to mammalian mt-rRNAs is significantly lower than that for bacterial and cytoplasmic rRNAs. There are 10 modifications identified to date in mt-rRNAs, including three 2′-O-ribose methylations, six base methylations, and one pseudouridylation (Figure 1 and Table 2). The majority of these modifications were identified around 40 years ago by Dubin and colleague in hamster cells (Dubin, 1974; Dubin and Taylor, 1978; Baer and Dubin, 1980, 1981). Since then, new modifications have been uncovered, and the enzymes responsible for all thus far identified modifications have been described. The roles of mitochondrial methyltransferases and their mt-rRNA targets are discussed below.

**Table 2:** Mitochondrial rRNA methyltransferases, corresponding bacterial homologs, and modified RNA residues.

*Homo sapiens* Enzyme	*H. sapiens* rRNA	Reference	*E. coli* Enzyme	*E. coli* rRNA	Reference
TFB1M	m^6^_2_A936 m^6^_2_A937	(Cotney and Shadel, 2006; Liu et al., 2019)	KsgA/RsmA	m^6^_2_A1518 m^6^_2_ A1519 16S rRNA	(Helser et al., 1972; Poldermans et al., 1979; Formenoy et al., 1994)
NSUN4	m^5^C841	(Metodiev et al., 2014)	RsmF	m^5^C1407 16S rRNA	(Demirci et al., 2010a)
TRMT2B	m^5^U429	(Laptev et al., 2019)	RlmD	m^5^U1939 23S rRNA	(Madsen et al., 2003; Auxilien et al., 2011)
METTL15	m^4^C839	(Haute et al., 2019)	RsmH	m^4^Cm1402 23S rRNA	(Kimura and Suzuki, 2010)
MRM1	Gm1145	(Lee et al., 2013; Lee and Bogenhagen, 2014)	RlmB	Gm2251 23S rRNA	(Lövgren and Wikström, 2001)
MRM2	Um1364	(Lee et al., 2013; Lee and Bogenhagen, 2014; Rorbach et al., 2014)	RlmE	Um2552 23S rRNA	(Caldas et al., 2000)
MRM3	Gm1370	(Lee et al., 2013; Lee and Bogenhagen, 2014; Rorbach et al., 2014)	No homolog		
TRMT61B	m^1^A947	(Bar-Yaacov et al., 2016)	TrmI	m^1^A58 tRNA	(Droogmans et al., 2003)

## Mitochondrial rRNA Methyltransferases

### mt-SSU Methyltransferases

#### TFB1M (m^6^_2_A936/m^6^_2_A937)

TFB1M is a homolog of the universally conserved methyltransferase KsgA (also known as RsmA). Structural analyses of bacterial KsgA in complex with the 30S subunit indicated that the enzyme binds to the inactive conformation of the SSU with helix 44 in a displaced conformation (O’Farrell et al., 2004; Boehringer et al., 2012). This binding blocks the interaction between helices 44 and 45, which form the decoding center in the mature ribosomal subunit. Once the 30S platform and helix 45 reach a near mature conformation, KsgA methylates helix 45, which leads to its dissociation. The release of KsgA is required for helix 44 to assume its native position in the 30S subunit and for 17S rRNA processing (Boehringer et al., 2012). KsgA’s role in the formation of the translationally active 30S subunit conformation may explain its conservation across all domains of life.

Initially, mitochondrial TFB1M was considered to function as a transcription factor alongside TFB2M. However, it was instead shown to be a dimethyltransferase responsible for m^6^_2_A modification of the 12S rRNA (Cotney and Shadel, 2006; Liu et al., 2019). This modification occurs within two adjacent adenines, A936 and A937 (human mtDNA position: m.1583A and m.1584A, respectively), located in the tetraloop “GGAA” of helix 45 at the 3′-end of the mt-12S rRNA, which is extremely conserved in both sequence and structure (McCulloch et al., 2002). TFB1M binds S-adenosylmethionine (SAM), the methyl-donating substrate of methyltransferase enzymes, and can functionally complement KsgA ablation by restoring the dimethylation of the conserved stem-loop (McCulloch et al., 2002; Seidel-Rogol et al., 2003).

Studies in the fly *Drosophila melanogaster* (Dm) demonstrated that the Dm-TFB1M ortholog is mainly involved in translation regulation, while Dm-TFB2M is involved in transcription (Matsushima et al., 2004, 2005). This is consistent with studies showing that human TFB1M has a greater rRNA methyltransferase activity compared to TFB2M (Cotney and Shadel, 2006). The importance of the m^6^_2_A modification has been subsequently highlighted in an *in vivo* study, showing that mouse *Tfb1m* knock-out is embryonic lethal, while heart conditional knock-out causes loss of mt-12S rRNA dimethylation, affecting the stability of the mt-SSU and leading to altered mitoribosome assembly and mitochondrial translation (Metodiev et al., 2009).

Recently, the crystal structure of TFB1M in complex with helix 45 and SAM has revealed its unique properties compared to its paralogue TFB2M (Liu et al., 2019). Notably, TFB1M has a clear acid-active pocket, which accommodates SAM, while the same region in TFB2M is highly positively charged, thus facilitating interaction with DNA molecules. Furthermore, A937 has been recognized as the first adenine to be methylated and G934 is necessary for this methylation, since it brings A937 into the active center where SAM is sheltered (Liu et al., 2019).

Interestingly, two mt-SSU assembly factors, the human ribosome-binding factor A (RBFA) and Era-like 12S mitochondrial rRNA chaperone 1 (ERAL1), have been identified to interact with the hairpin at the 3′-terminus of mt-12S rRNA, where dimethylation by TFB1M occurs (Dennerlein et al., 2010; Uchiumi et al., 2010; Rozanska et al., 2017). RBFA was observed to bind directly the dimethylation site of mt-12S rRNA and *RBFA* knock-down resulted in a reduced level of modification, suggesting that RBFA helps to expose adenines for subsequent methylation by TFB1M (Rozanska et al., 2017). Future studies are necessary to describe the molecular details of RBFA involvement in this process.

#### TRMT2B (m5U429)

Although methyl-5-uridine (m^5^U) is one of the most abundant RNA modifications (Boccaletto et al., 2018), it remains poorly characterized. Bacterial RlmD, which modifies m^5^U1939 of the 23S rRNA in *E. coli*, is considered to be the ancestral m^5^U RNA methyltransferase (Auxilien et al., 2011). Due to gene duplication and specialization, RlmC and TrmA enzymes have evolved in addition to RlmD. RlmC modifies m^5^U747 of the 23S rRNA (Madsen et al., 2003), and TrmA introduces m^5^U at position 54 in the T-loop of several tRNAs (Ny and Björk, 1980). In *S. cerevisiae*, Trm2 catalyzes this tRNA modification (Nordlund et al., 2000). Sequence homology analysis identified two mammalian proteins, TRMT2A and TRMT2B, as m^5^U methyltransferase candidates (Carter et al., 2019). TRMT2A was identified as the enzyme responsible for m^5^U54 in the cytosol (Carter et al., 2019; Powell and Minczuk, 2020), while TRMT2B was suggested to methylate tRNAs in mitochondria (de Crécy-Lagard et al., 2019).

A recent study by Laptev et al. showed that *Trmt2b* knock-out in mouse cells leads to a lack of m^5^U425 methylation (mouse numbering, equivalent to human m^5^U429, mtDNA position: m.1076T) in mt-12S rRNA as well as of U54 in certain mitochondrial tRNAs, indicating that TRMT2B might act as a dual tRNA/rRNA methyltransferase (Laptev et al., 2019). At the same time, Powell and Minczuk showed that TRMT2B is located in human mitochondria and plays an essential role in methylation of both tRNAs and 12S rRNA (Powell and Minczuk, 2020). Similar to yeast Trm2 (Nordlund et al., 2000), no apparent impairment of mitochondrial tRNA stability, mitoribosome integrity, or mitochondrial protein synthesis was detected upon TRMT2B loss in human cells (Powell and Minczuk, 2020). Interestingly, while the rate of protein synthesis was also not affected in mouse *Trmt2b* knock-out model, a small, but statistically significant, decrease in the activity of OxPhos complexes I, III, and IV was detected, which may be explained by a reduction of protein synthesis fidelity (Laptev et al., 2019).

The mild phenotype upon TRMT2B loss is in contrast to a detrimental effect on mitochondrial translation observed upon the loss of other mt-rRNA modifying enzymes, including for example TFB1M (Metodiev et al., 2009) or NSUN4 (Metodiev et al., 2014). The exact role of the modification introduced by TRMT2B and its contribution to mitochondrial function in different environmental conditions and/or specific tissues requires further investigation.

#### NSUN4 (m^5^C841)

NSUN4 is a mitochondrial rRNA methyltransferase that belongs to the m^5^C methyltransferase family and introduces m^5^C911 modification of mt-12S rRNA in mice (human m^5^C841, mtDNA position: m.1488C; Metodiev et al., 2014).

In *Thermus thermophilus*, the corresponding residue (position C1404) is modified by the methyltransferase RsmF, which also modifies C1400 and C1407. All three m^5^C residues modified by RsmF in *T. thermophilus* 16S rRNA are clustered around the decoding center, close to sites of contact with tRNA, mRNA, and elongation factor G. The *T. thermophilus* RsmF null mutants were shown to be thermosensitive. *In vitro*, RsmF methylates C1404 to around 35% with naked 16S rRNA as a substrate and to 100% in the context of 30S subunit, suggesting that this modification is likely to be introduced at the later stages of SSU biogenesis (Demirci et al., 2010a)

In mice, knock-out of *Nsun4* results in defective embryonic development, while heart conditional knock-out causes an OxPhos impairment, leading to severe cardiomyopathy (Metodiev et al., 2014). Sucrose gradient centrifugation analysis revealed that NSUN4 ablation leads to the accumulation of free mt-SSU and mt-LSU, preventing monosome formation (Metodiev et al., 2014).

Interestingly, NSUN4 has been shown to form a stable heterodimeric complex with MTERF4 that is targeted to the mt-LSU and plays an essential role in mt-LSU assembly, independent of the methylation activity of NSUN4 (Cámara et al., 2011). NSUN4 lacks RNA binding domains; instead, structural studies revealed that a positively charged surface forms an RNA binding path from MTERF4, along NSUN4, all the way into its active site, suggesting that both proteins contribute to RNA recognition (Spahr et al., 2012). *In vitro* methylation experiments showed that MTERF4 strongly stimulates the specificity of NSUN4; however, the monomeric NSUN4 is still able to methylate the substrate albeit with lower specificity (Yakubovskaya et al., 2012).

#### METTL15 (m^4^C839)

Recently, METTL15, from the methyltransferase-like (METTL) family, was reported to be involved in m^4^C839 (human mtDNA position: m.1486C) modification of human 12S rRNA (Haute et al., 2019; Chen et al., 2020). An equivalent position in bacteria (C1402) has two modifications, N^4^ and 2′-O-methylations (m^4^Cm), introduced by RsmH and RsmI, respectively (Kimura and Suzuki, 2010), while in the human mitoribosome 2′-O-methylation at C839 is not conserved. *In vitro*, recombinant *E. coli* RsmH and RsmI reconstitute m^4^Cm1402 on the 30S subunit, but not on the naked 16S rRNA, suggesting that these modifications are formed at a late step during 30S assembly. Moreover, RsmH prefers 2′-O-methyl cytosine as a substrate and, therefore, m^4^C in bacteria likely occurs subsequent to the 2′-O-methylation. Modified m^4^Cm1402 interacts directly with the P-site codon of the mRNA and the lack of *N* ^4^ methylation increases the efficiency of non-AUG initiation and decreases the rate of UGA read-through, implying that m^4^Cm1402 plays a role in fine-tuning the ribosomal decoding center, thus increasing decoding fidelity (Kimura and Suzuki, 2010).

In human cells, METTL15 localizes to mitochondria, and the lack of this enzyme leads to mitochondrial dysfunction. METTL15 was shown to interact with the mt-SSU, and knock-out of *METTL15* results in significantly decreased m^4^C839 levels in 12S rRNA (Haute et al., 2019; Chen et al., 2020). Loss of m^4^C839 modification leads to aberrant assembly of the mt-SSU and accumulation of late-stage assembly intermediates, suggesting an important role of this modification in the 12S rRNA folding and, consequently, interaction with the mitoribosomal proteins. Importantly, both published reports detected reduction in the m^5^C841 modification catalyzed by NSUN4 (Metodiev et al., 2014) concomitant to decreased m^4^C839 modification, revealing a potential crosstalk between modifications of these two nearby residues.

Interestingly, Shi et al. have recently identified another member of the METTL family, METTL17, to modulate m^4^C839 modification (Shi et al., 2019). METTL17 localizes to mitochondria and associates with the mt-SSU. Loss of METTL17 leads to around 70% reduction of m^4^C840 and 50% reduction of m^5^C842 of 12S mt-rRNA, severely compromising integrity of the mt-SSU and mitochondrial protein translation (Shi et al., 2019). Collectively, these data suggest an important role for METTL17 in mitochondrial function, although further work is needed to assess potential interdependence of METTL15 and METTL17 in m^4^C839 methylation.

### mt-LSU Methyltransferases

#### MRM1 (Gm1145)

Human mitochondrial 16S rRNA contains three 2′-O-ribose methylation sites: Gm1145, Um1364, and Gm1370 (human mtDNA positions: m.2815G, m.3039T, m.3040G, respectively). These methylations reside in highly conserved sites found within the peptidyl transferase center (Decatur and Fournier, 2002).

The peptidyl transferase region of 16S rRNA involved in the binding of tRNA in the P-site (referred to as peptidyl-transferase loop, P-loop) undergoes 2′-O-ribose methylation at G1145 by MRM1. This modification is highly conserved across ribosomes of different species and seems to play a direct role in peptidyl-tRNA recognition (Sergiev et al., 2018). In yeast, the equivalent modification, Gm2270, on mitochondrial 21S rRNA is catalyzed by Pet56p/MRM1 (Sirum-Connolly and Mason, 1993). The loss of Pet56p in *S. cerevisiae* leads to a defect in the maturation of the mt-LSU with an accumulation of slower sedimenting particles by sucrose gradient (Sirum-Connolly and Mason, 1993). Interestingly, a variant of Pet56p with an amino acid substitution in the SAM pocket that abolishes its methyltransferase activity does not alter the formation of fully functional mitoribosomes (Lövgren and Wikström, 2001). This suggests that the role of Pet56p in ribosome assembly is independent of its methyltransferase activity.

In bacteria, Gm2251 of 23S rRNA is catalyzed by RlmB (Lövgren and Wikström, 2001). The crystal structure of RlmB revealed the presence of an N-terminal domain connected *via* a linker to a catalytic C-terminal domain, responsible for the dimerization of RlmB in solution (Michel et al., 2002). A strong similarity between the N-terminal domain and the ribosomal proteins L7 and L30 was observed; in particular, the presence of conserved residues that are essential for binding of L30 to RNA suggested that the N-terminal domain might be important for RlmB interaction with the 23S rRNA (Michel et al., 2002). Interestingly, in contrast to Pet56p, no effect on growth rate or ribosome assembly was observed upon RlmB depletion (Lövgren and Wikström, 2001).

Human methyltransferase MRM1 was shown to localize in mitochondria in close proximity to mtDNA nucleoids (Lee et al., 2013) and was found to co-sediment with the mt-LSU through gradient sedimentation experiments. Primer extension and DNAzyme-mediated RNA cleavage assays were used to assign the 2′-O-ribose methylation of Gm1145 to MRM1 (Lee et al., 2013; Lee and Bogenhagen, 2014). Further studies are essential to understand the role of MRM1 and Gm1145 modification in mitoribosome biogenesis and function.

#### MRM2 (Um1369)

MRM2 is a uridine 2′-O-methyltransferase that modifies U1369 position of the mitochondrial 16S rRNA. This highly conserved modification is located in the peptidyl transferase center and is implicated in the interaction of the ribosome with an aminoacyl(A)-site tRNA. Human MRM2 is closely related to yeast MRM2p and bacterial FtsJ/RlmE (Lee et al., 2013). Both MRM2p and FtsJ/RlmE have been extensively studied and their ablation has been shown to lead to severe growth defects and thermosensitive phenotypes (Caldas et al., 2000; Pintard et al., 2002b).

In *S. cerevisiae* mitochondria, Mrm2p was shown to co-sediment with 21S rRNA by sucrose gradient centrifugation analysis and to methylate, both *in vitro* and *in vivo*, U2791 of 21S rRNA in the context of the LSU, but not naked rRNA (Pintard et al., 2002a). Alignment analysis with its putative bacterial ortholog FtsJ/RlmE showed high similarities between the two proteins. The *ftsJ* gene in *E. coli* was originally identified as a heat-inducible gene (Richmond et al., 1999), and subsequently FtsJ was shown to be a SAM-dependent methyltransferase responsible for 2′-O-methylation of U2552 in 23S rRNA (Caldas et al., 2000).

RlmE depletion was shown to cause striking defects in the ribosome assembly process, leading to an accumulation of intermediates of the 30S and 45S particles, and a decrease of the 70S particles and polysomes (Bügl et al., 2000; Caldas et al., 2000; Arai et al., 2015). Initially, RlmE was thought to methylate the 23S rRNA in the context of the 50S subunit rather than the 45S intermediates that accumulate upon its depletion (Bügl et al., 2000). However, it was later shown that 45S, the precursor of the 50S subunit, is the real substrate of RlmE and that methylation of U2552 triggers the formation of the 50S subunit (Arai et al., 2015). Intriguingly, expression of two GTPases, EngA and ObgE, restored the defective phenotypes caused by RlmE ablation despite the absence of the U2552 modification, suggesting an interesting link between GTPase activity and RNA methylation (Tan et al., 2002).

As for MRM1, primer extension assay and DNAzyme-mediated RNA cleavage analysis allowed to identify MRM2 to be responsible for modification of Um1369 (Lee et al., 2013; Lee and Bogenhagen, 2014; Rorbach et al., 2014). Silencing of *MRM2* in cultured human cells led to decreased mitochondrial translation and OxPhos impairment, while immunoprecipitations and sucrose gradient centrifugation analyses revealed an interaction between MRM2 and the mt-LSU (Rorbach et al., 2014). Upon *MRM2* silencing, the steady-state levels of mt-LSU were found to be decreased, without affecting the mt-SSU levels, confirming that Um1369 is important for the biogenesis of the mt-LSU. Furthermore, *MRM2* downregulation resulted in a partial decrease in Gm1370 modification, alongside Um1369, with the former modification being introduced by MRM3. This suggests an interdependence between methylation of U1369 and G1370 and implies that MRM2 may act at an earlier stage of mitoribosome biogenesis than MRM3.

#### MRM3 (Gm1370)

MRM3 is responsible for methylation of G1370, adjacent to Um1369 in the A-loop of the mt-LSU. The equivalent residue in *E. coli* 23S, G2553, pairs with C75 of the aminoacyl tRNA in the bacterial ribosomal A-site (Kim and Green, 1999). In *E. coli*, G2553 is not modified, and neither is the yeast equivalent in mitochondrial 21S rRNA (G2792). Interestingly, yeast cytosolic LSU 25S rRNA has 2′-O-methylguanosine modifications in the analogous site (Gm2922) introduced by nucleolar protein Spb1p (Kressler et al., 1999b). Modification of G2922 is a late event occurring on the 27S ribosome intermediate and is essential for ribosome biogenesis (Lapeyre and Purushothaman, 2004). In human cytoplasmic 28S rRNA, the corresponding site is G4499 and it is 2′-O-methylated *via* a snoRNA-guided mechanism (Sergiev et al., 2018).

Human MRM3 associates with the mt-LSU, as revealed by co-immunoprecipitation and sucrose gradient sedimentation analyses (Lee et al., 2013). *MRM3* silencing reduced Gm1370 methylation and, consequently, mitochondrial translation and OxPhos function. Moreover, downregulation of MRM3 expression resulted in the accumulation of species consistent with mt-LSU pre-ribosomal particles, suggesting that methylation of G1370 likely occurs during the late-stage of mitoribosome assembly (Rorbach et al., 2014).

#### TRMT61B (m^1^A947)

TRMT61B is a dual function methyltransferase that modifies both mt-tRNA (Chujo and Suzuki, 2012) and mt-rRNA (Bar-Yaacov et al., 2016). The conserved residues of its bacterial homolog, TrmI, are well characterized for their catalytic function (Barraud et al., 2008) and contribution to binding of SAM (Dégut et al., 2016). TrmI is responsible for SAM-dependent N^1^-methylation of adenosine 58 in the T-loop of many tRNAs and its inactivation in the hyperthermophilic bacterium *T. thermophilus* results in a thermosensitive phenotype (Droogmans et al., 2003).

Initially, TRMT61B was found to act as a mitochondrial tRNA methyltransferase responsible for m^1^A58 of tRNA^Leu(UUR)^, tRNA^Lys^, and tRNA^Ser(UCN)^ (Chujo and Suzuki, 2012). However, a more recent study identified TRMT61B as the enzyme responsible for the m^1^A modification at position 947 (mtDNA: m.2617A) of the mt-16S rRNA (Bar-Yaacov et al., 2016). This was supported by siRNA experiments coupled with primer extension assay and RNA sequencing analyses showing a hypomethylation of m^1^A947 upon TRMT61B depletion. *In vitro* methylation assays further confirmed the ability of TRMT61B to modify naked mt-16S rRNA, suggesting a possible role for TRMT61B in the early stages of mt-LSU maturation (Bar-Yaacov et al., 2016).

The m^1^A947 modification of the 16S rRNA occurs in most vertebrates and is enriched in the mature mammalian mitoribosome (Bar-Yaacov et al., 2016). Mapping of m^1^A947 into the 55S monosome structure revealed that the modification is located in helix 71 of the mt-LSU, in proximity to the intersubunit bridge B3, where interaction with the mt-SSU occurs (Figure 1). Phylogenetic studies show that this region is structurally conserved in bacterial and cytoplasmic ribosomes, where the same position is evolutionarily occupied by an unmodified guanine and an unmodified uracil, respectively (Yusupova and Yusupov, 2014; Greber et al., 2015; Noeske et al., 2015).

In mitoribosomes, helix 71 seems to form an interdomain interaction with helix 92 and to stabilize a tertiary interaction with helix 64 *via* an electrostatic bond. Notably, *in vivo* substitution of the unmodified guanine in bacterial ribosomes with an unmodified adenine led to an alteration of protein synthesis and slower growth rates, while no effect was detected in the presence of an unmodified uracil (Bar-Yaacov et al., 2016). This data corroborates the hypothesis that methylation of A947 is essential for the maintenance and stabilization of the mitoribosome structure, as the unmodified adenine lacks the positive charge needed to bind the negatively charged backbone of helix 64. In contrast, in bacteria, the unmodified guanine can interact with the 23S rRNA *via* a hydrogen bond, while in the cytoplasm the unmodified uracil can interact *via* a water molecular bridge with the rRNA. It is intriguing to notice how vertebrate mitochondrial ribosomes diverged from their bacterial ancestors by replacing an unmodified nucleotide with an rRNA modification that requires the recruitment of a nuclear-encoded rRNA methyltransferase. Although the exact function of m^1^A947 still needs to be elucidated, it is clear that this modification is important for the stabilization of the mitoribosome structure.

### Mitochondrial rRNA Methyltransferases and Disease

Our current knowledge of the pathological role of mitochondrial rRNA-modifying enzymes is limited. A patient manifesting symptoms of mitochondrial encephalopathy, lactic acidosis, and stroke-like episodes (MELAS) syndrome was found to carry a mutation in *MRM2* (Garone et al., 2017). While the patient fibroblasts did not exhibit the same phenotypes ascertained in *MRM2* knock-down experiments (Rorbach et al., 2014), complementation of the *MRM2* knock-out yeast model with the patient MRM2 variant could not rescue the respiration defect detected, thus supporting the pathogenicity of *MRM2* mutation in MELAS syndrome (Garone et al., 2017). To date, MRM2 is the only rRNA modifying enzyme in mitochondria with a pathogenic mutation directly linked to a primary mitochondrial disorder.

TFB1M was initially linked to aminoglycoside antibiotic-induced deafness because studies using TFB1M transgenic mice showed activation of pro-apoptotic factor E2F1 caused by TFB1M-hypermethylation of mt-12S rRNA (Raimundo et al., 2012). However, patients carrying the mt-DNA mutation m.A1555G, previously identified as a cause of deafness and located in proximity to the two adenines methylated by TFB1M, did not manifest changes in mt-12S rRNA methylation levels compared to controls, thus putting into question the role of TFB1M in the pathogenesis of this disorder (O’Sullivan et al., 2015). Interestingly, another study found a common variant of *TFB1M* to be associated with reduced insulin secretion and increased risk of type 2 diabetes in Tfb1m-deficient mice (Koeck et al., 2011). Similar observations were documented for a mouse model with beta cell-specific knock-out of *Tfb1m* that resulted in lower insulin secretion, mitochondrial dysfunction, and eventual development of type 2 diabetes (Sharoyko et al., 2014).

TRMT61B transcript expression was altered in total RNA extracted from astrocytes of Alzheimer’s disease patients compared to controls (Sekar et al., 2015). In a separate study, functional and expression quantitative trait loci analyses linked TRMT61B to estrogen receptor-negative breast cancer (Couch et al., 2016). Further research is needed to clarify the potential role of TFB1M or TRMT61B, as well as other rRNA modifying enzymes, in human disease.

## Future Prospects: Emerging Technologies to Investigate rRNA Methylation

Although there has been significant progress in the detection of mt-RNA modifications and corresponding enzymes, the complete landscape of mt-rRNA methylations and the specific roles of these modifications remain to be fully elucidated. Several cytosolic rRNA modifications exist at very low levels and only recent technical advances have enabled their detection (Taoka et al., 2018). Furthermore, there is increasing evidence that ribosomal modifications are dynamic, and their levels can be regulated under different physiological conditions (Erales et al., 2017). Further studies are needed to assess if the same is true for mt-rRNA modifications.

Due to the numerous types of RNA modifications, there is no universal technique to identify all of them simultaneously. Some recent approaches include nuclease protection assays and reversed-phase high-performance liquid chromatography (Yang et al., 2016) or sequencing profiling to measure reverse transcriptase drop-off rates coupled to mass spectrometry (Enroth et al., 2019). Transcriptome-wide next-generation sequencing and mass spectrometry methods have also been used to estimate the abundance of individual RNA modifications (Zhang et al., 2019). Alternative approaches use immunoprecipitation and next-generation sequencing in pooled samples to gain insights into the stoichiometry of modified nucleotides while preserving sequence information (Dominissini et al., 2013). Additionally, emerging technologies aim to detect RNA modifications at the single-cell level (Ranasinghe et al., 2018).

Among the 172 RNA modifications reported to date, more than 40% involve the methyl-group (Boccaletto et al., 2018). Recent reviews have highlighted various approaches, including immunochemical, methylation-sensitive enzymes, hybridization, and high-throughput sequencing technologies to identify specific RNA methylations (Li et al., 2016; Ovcharenko and Rentmeister, 2018), while biochemical approaches have enabled mapping of modifications that do not interfere with Watson–Crick base pairing, including m^6^A (Hartstock et al., 2018). Furthermore, a transcriptome-wide, single-base resolution method based on the modification of RNA bisulfite sequencing was reported to simultaneously detect m^5^C, pseudouridylations, and m^1^A modifications (Khoddami et al., 2019).

Despite these advancements, the aforementioned techniques often do not cover the mt-RNA modifications efficiently, partially due to the lower abundance of mt-RNA in comparison to the cytosolic RNA pool. Nevertheless, the suitability of these methodologies for mitochondrial studies is promising, and the enrichment of mitochondria by standard isolation methods from cultured cells or tissues (for example, Mercer et al., 2011) can be introduced in the adapted protocols to yield explicit and deeper coverage on mt-RNA modifications.

Advances in the field of cryoEM have significantly contributed to the characterization of ribosomes, providing structural insights at atomic resolution. Progress in the cryoEM field has enabled the detection of rRNA modifications by detecting the positions of extra densities in electron density maps (Liu et al., 2017). Recently, a high-resolution cryoEM 3D structure of the human 80S ribosome identified 136 rRNA modification sites, including 60 2′-O methylations, 25 pseudouridylation sites, and 51 other base modifications, all located in or close to functionally important sites within the ribosome (Natchiar et al., 2017). Some discrepancies between the data obtained by cryoEM studies (Natchiar et al., 2017) and other quantitative techniques detecting modifications (Taoka et al., 2018) illustrate the need for complementary techniques to elucidate the entire epitranscriptome map of rRNA modifications.

**Figure 2:**
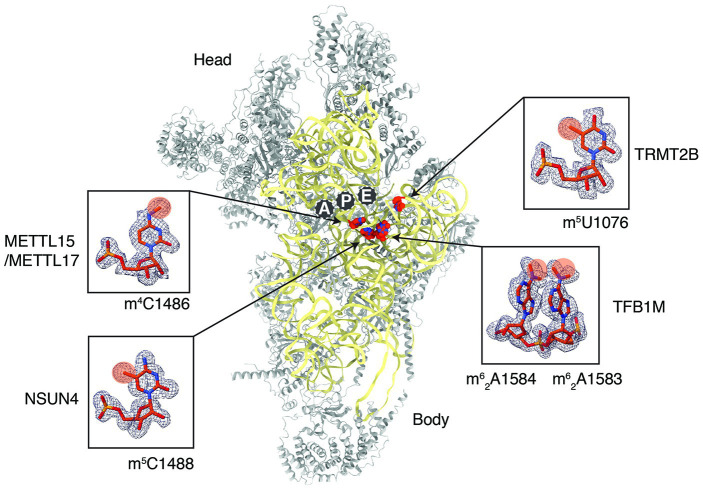
Methylated 12S mitoribosomal rRNA (mt-rRNA) residues in the human mitoribosomal small subunit as revealed by cryoEM. The structure of human 28S (PDB: 6RW4) reveals the distribution of 12S rRNA methylation sites (red). The proteins of the 28S are colored gray and the 12S mt-rRNA is in yellow. The zoom-in panel displays the methylated rRNA residues with corresponding density maps. Chemical groups that are added enzymatically through the action of specific enzymes are highlighted in orange.

Our preliminary analysis of the already available high-resolution maps of mt-SSU (Khawaja et al., 2020) allowed us to identify densities corresponding to all five methylations of the 12S rRNA (Figure 2), proving that cryoEM is indeed a great tool to investigate mt-rRNA modifications. There is no doubt that the same will be achieved soon for 16S rRNA and can be expanded to mitoribosomes isolated from different tissues, thanks to the continuous improvements in cryoEM methodology.

As mitochondria are a central organelle critical for a variety of cellular processes, an in-depth understanding of RNA modifications, including methylation within mitochondria, will improve our understanding of mitochondrial gene expression regulation and its link to human pathophysiology.

## Author Contributions

ML and JR designed and coordinated the writing of the manuscript. AK prepared the figures. ML, JR, MC, and SG wrote the manuscript. All authors contributed to the article and approved the submitted version.

## Conflict of Interest

The authors declare that the research was conducted in the absence of any commercial or financial relationships that could be construed as a potential conflict of interest.
